# Biogenic amine modulation of honey bee sociability and nestmate affiliation

**DOI:** 10.1371/journal.pone.0205686

**Published:** 2018-10-25

**Authors:** Susie E. Hewlett, Jacqueline D. Delahunt Smoleniec, Deborah M. Wareham, Thomas M. Pyne, Andrew B. Barron

**Affiliations:** 1 Department of Biological Sciences, Macquarie University, Sydney, New South Wales, Australia; 2 Department of Health Professions, Macquarie University, Sydney, New South Wales, Australia; RMIT University, AUSTRALIA

## Abstract

Biogenic amines modulate a range of social behaviours, including sociability and mechanisms of group cohesion, in both vertebrates and invertebrates. Here, we tested if the biogenic amines modulate honey bee (*Apis mellifera*) sociability and nestmate affiliation. We examined the consequences of treatments with biogenic amines, agonists and antagonists on a bee’s approach to, and subsequent social interactions with, conspecifics in both naturally hive-reared bees and isolated bees. We used two different treatment methods. Bees were first treated topically with compounds dissolved in the solvent dimethylformamide (dMF) applied to the dorsal thorax, but dMF had a significant effect on the locomotion and behaviour of the bees during the behavioural test that interfered with their social responses. Our second method used microinjection to deliver biogenic amines to the head capsule via the ocellar tract. Microinjection of dopamine and a dopamine antagonist had strong effects on bee sociability, likelihood of interaction with bees, and nestmate affiliation. Octopamine treatment reduced social interaction with other bees, and serotonin increased the likelihood of social interactions. HPLC measurements showed that isolation reduced brain levels of biogenic amines compared to hive-reared bees. Our findings suggest that dopamine is an important neurochemical component of social motivation in bees. This finding advances a comparative understanding of the processes of social evolution.

## Introduction

The biogenic amines (including dopamine, serotonin, octopamine) are involved in many types of behaviour, including various social behaviours [1–2]. In mammals, mechanistic studies of sociability (tendency to aggregate [3]) and affiliation have been most extensively studied in prairie voles (*Microtus ochrogaster*), as they form life long partnerships and coparent [4]. Part of the prairie vole partnership formation process involves learning the olfactory signature of the partner and associating the olfactory cue with reward [5]. Inhibiting the dopaminergic mesolimbic reward system inhibits a partner preference developing [5]. In both male and female voles, activation of the D2 dopamine receptor in the nucleus accumbens is key to partner preference formation [6–9], as well as dopamine (DA) activity in the basal ganglia [10]. Similarly, increases in DA levels and dopaminergic neuron activity have been linked to bird partner preference formation [11], and also correlate with flocking behaviours of larger groups [12–14]. O’Connell and Hofmann [15] argue that the control of social behaviour by the coordinated activity of the reward system and the ‘social behaviour brain network’–proposed by Newman [16]–is applicable to all vertebrate lineages. Even more recently, the DA-dense striatum of the basal ganglia has been hypothesised as key to the evolution of human empathy and altruism [17]. Here, we test for biogenic amine involvement in honey bee (*Apis mellifera*) sociability and nestmate affiliation.

Whilst DA signaling is a major component of the mammalian reward system [18], both octopamine (OA) and DA signaling are involved in the insect reward learning circuitry [19–22]. Furthermore, OA, DA and serotonin (5-hydroxytrptamine, 5HT) have been implicated in an array of eusocial insect behaviours, including group organization and coordination, pheromonal communication, social recognition and resultant interactions [23–33]. For example, OA systems are involved in honey bee division of labour [23–25], and coordinated defensive action of honey bees may involve 5HT [26]. Although OA is strongly linked to both honey bee and ant (*Camponotus fellah*) social recognition [27–28], DA and 5HT have also been shown to have modulatory effects on degree of social recognition [29–30]. Similarly, DA is involved in pheromonal communication between honey bee workers and their queen [31–32], whereas brain OA levels were regulated by the queen pheromone in an ant (*Solenopsis invicta*) [33]. The highly similar pharmacological properties of DA and OA receptors [34] may explain their shared functional roles. Hence, we expect the biogenic amines to play a role in the preferential affiliation to nestmates of bees, as this choice requires the ability to discriminate between conspecifics.

The biogenic amines are also involved in the development and maintenance of sociable phenotypes in migratory and desert locusts (*Locusta migratoria* and *Schistocerca gregaria*, respectively) [35–42]. Mechanosensory information from touching conspecifics causes these locusts to switch from a solitary to a gregarious state [43]. In the absence of triggering sensory inputs, injection of 5HT into the peripheral nervous system initiates the sociable state in *S*. *gregaria* [35]. Persistent sociable states in *S*. *gregaria* coincide with reduced DA and 5HT levels compared to solitary conspecifics [36], whereas *L*. *migratoria* have sustained high DA levels during the sociable state [37]. Crowding increases OA brain levels in both *S*. *gregaria* and *L*. *migratoria* [36, 38], and the activity of specific receptor subtypes for both OA and DA have been implicated in modulating locust solitary-sociable states [38–42]. These studies show that for locusts, biogenic amine systems control the social approach response and the level of sociability expressed. This behaviour is important as it determines whether a group forms or not, and a group is a prerequisite for social interactions. Sociability is also a readily comparable social behaviour throughout the animal kingdom. Based on the locust findings, we predict that the biogenic amines will have an effect on honey bee sociability.

In vertebrate studies, the level of sociability and strength of affiliations are assessed with two-choice preference assays [44–46]. Recently, a bioassay comparable to vertebrate and locust preference assays was developed for eusocial insects [47] and revealed that honey bees are highly sociable and prefer to remain in proximity to nestmates rather than non-nestmates when given a choice. Rearing animals in isolation and later testing them in assays that measure their sociability and preferential affiliations has revealed the influence of the developmental environment on vertebrate sociability and subsequent social behaviours [48–49]. Recently, it was found that honey bee sociability and affiliation is also experience-dependent, but high levels of sociability could be reinstated by brief exposure to the hive environment even after 5 days of age [47]. Here we combine the insect assay with pharmacological treatments to test for biogenic amine involvement in bee sociability and nestmate affiliation.

We used two methods to apply treatments to the brain. Topical application using the solvent dimethylformamide (dMF) was used as it has previously been shown to effectively deliver biogenic amines to the honey bee brain, but is less invasive than injections [50]. We also microinjected the biogenic amines and antagonists to the brain via the ocellar tract. Although more invasive as the lens of the median ocellus must be removed for injection, the solvent is a bee-specific saline solution [51]. Based on the developmental findings [47], we treated 5-day-old bees with DA, OA, 5HT, an agonist or antagonist, and tested their sociability and nestmate affiliation. We treated and tested both isolated and naturally raised honey bees to investigate if biogenic amine receptor activation or inhibition is important in the initiation of approach and aggregation in socially naïve bees, as has been reported in locusts [36], or modulates these behaviours that have already developed in socially experienced bees.

## Materials and methods

### Subjects

Bees from three honey bee colonies were used, taken from the fauna park at Macquarie University, Sydney, Australia. Frames containing eclosing brood (nibbling their cell cap away) were pulled from the hive and brushed free of any adult bees. For hive-reared bees, frames were placed in a dark incubator at 33°C for several hours. All the newly emerged bees were collected into a container and individually painted on the thorax (uniPOSCA, Mitsubishi pencil co. UK) for later identification, and returned to their natal colony.

For the isolation treatment, newly emerging bees, from frames brushed free of any already emerged bees, were gently removed from their cell and placed individually in a falcon tube and maintained in a dark incubator at 33°C. They were fed 40% honey-water daily and had no direct contact with other bees as adults since their cell was capped during the larval stages. Although bees as young as 5 days old consume pollen as well as honey, no difference in assay performance was found for isolated bees raised on different diets [47], hence we are confident the honey diet is sufficient for bees in this assay. All bees were tested at 5 days old.

The same honey bee colonies from the fauna park at Macquarie University were used for both topical and ocellar treatment experiments, although at least one became queenless and the queen was replaced. Nonetheless, the same rearing and foraging environments were experienced by the bees in the two experiments. The rearing procedures for hive-reared and isolated bees were also identical in both the topical and ocellar experiments.

### Topical treatment via the dorsal thorax

Each testing day isolated bees were collected from the incubator and the hive-reared bees were collected from their hive, and all were transported individually in holding tubes (50ml Falcon tubes). All bees, except those assigned to the control group, were immobilised at 4°C so they could be harnessed without injury into metal holders (usually used in probiscus extention reflex (PER) experiments)[52]. Harnessed bees could freely move their antenna and mouthparts, and their dorsal thorax was exposed for topical treatment application. 10–15 minutes after being harnessed, each bee was tested for their response to honey and allowed to feed for up to 30 seconds. Approximately 5 minutes after feeding, bees were treated topically by applying 1μl of the biogenic amine treatment dissolved in dimethylformamide (dMF) to the dorsal thorax with a glass microcapillary pipette (Drummond Scientific, USA). All the bees were randomly assigned to a treatment and only tested once (**Table 1**). Treatments were either OA (1mg/ml), epinastine (OA antagonist, 1mg/ml), (+/-)-2-Amino-6,7- dihydroxy-1,2,3,4-tetrahydronaphthalene hydrobromide (6,7-ADTN, DA agonist [53–54], 2mg/ml) or fluphenazine (DA antagonist, 2mg/ml) and all dissolved in dMF. Chemicals were purchased from Sigma-Aldrich, Australia, except 6,7-ADTN which was purchased from Abcam, Australia. Vehicle-controls received 1μl of dMF, sham-controls were immobilised and harnessed but received no treatment, and controls were transferred directly from their holding tube to the test arena. Concentrations and volumes used are based on previous studies [50, 55]. The agonist 6,7-ADTN was used in place of DA as it amplified DA effects on sucrose response [56] and DA may be unstable in dMF.

**Table 1:** Sample size for each topical rearing-treatment group tested and used for behavioural analysis. Numbers in brackets are the number removed from the data set for spending more than 1 min on their back during the 5 min test.

Treatment	Hive-reared		Isolated	
	Full	>1 min on back removed	Full	>1 min on back removed
***Sham control***	14	14	17	12 (-5)
***Vehicle control***	14	14	14	5 (-9)
***Control***	18	18	14	12 (-2)
***6*,*7-ADTN***	26	26	29	19 (-10)
***Fluphenazine***	25	24 (-1)	25	18 (-7)
***OA***	24	24	22	15 (-7)
***Epinastine***	26	26	26	18 (-8)

### Injection via the ocellar tract

Each testing day bees were collected as above, and all bees (except those assigned to the control group), were also immobilised at 4°C so they could be harnessed without injury. Once harnessed, bees to be injected were allowed to fully awaken and offered a few seconds to feed on honey. Next the holder was fastened to the dissecting microscope (Olympus) platform using plasticine and the lens of the median ocellus was gently flicked up using a razor blade [52]. A 1μl Hamilton *7001* syringe was then loaded with 500nl of treatment and moved to the ocellus opening using a manual micromanipulator (Marishige, Japan). All the bees were randomly assigned to a treatment and only tested once (**Table 2**). Treatments were either DA (1mM / 0.2mg ml^-1^), fluphenazine (DA antagonist, 2mM / 1mg ml^-1^)[55], OA (1mM / 0.2mg ml^-1^), epinastine (OA antagonist, 3.9mM / 1.1mg ml^-1^)[57], 5HT (5HT, 1mM / 0.2mg ml^-1^) or a combination of ketanserin and methiothepin (5HT antagonists (KM), 0.5mM + 0.5mM / 0.2723mg ml^-1^ + 0.23mg ml^-1^)[35]. All were dissolved in bee-specific saline solution [51]. Chemicals were purchased from Sigma-Aldrich, Australia. Concentrations were based on previous studies (DA and OA systems [58] and 5HT system [35]) and 300nl was injected into the brain via the ocellar tract. Saline-controls received 300nl of bee saline only, sham-controls were immobilised and harnessed and their lens removed and replaced, and controls were transferred directly from their holding tube to the test arena. All treatment groups were tested approximately 30 minutes after the ocellus lens was replaced. Approximately 10 minutes prior to testing, bees were allowed to feed on honey offered on a toothpick for up to 30 seconds or until satiated to prevent hunger effects. Stimulus bees were also fed honey every 45 minutes for the same reason.

**Table 2:** The number of bees tested, removed for behavioural testing and available brain data for ocellar injection. Bold numbers were used in behaviour and brain analyses, and grey italics were sampled but not used in BA brain titer analyses.

		control	sham	saline	DA	OA	5HT	FLU	EPI	KM
Hive-reared	Total tested	18	14	14	16	15	16	16	15	15
	on back >1 min	0	0	0	0	0	0	0	0	0
	Behaviour n	**18**	**14**	**14**	**16**	**15**	**16**	**16**	**15**	**15**
	Brain n	**18**	**13**	**14**	**15**	**14**	**16**	*16*	*14*	*15*
Isolated	Total tested	20	19	20	17	20	19	na	na	na
	on back >1 min	2	4	4	2	5	3	na	na	na
	Behaviour n	**18**	**15**	**16**	**15**	**15**	**16**	na	na	na
	Brain n	**19**	**18**	**19**	**17**	**20**	**19**	*na*	*na*	*na*

### Sociability and nestmate affiliation test

Each bee was tested individually, and the order of testing was randomized each day to prevent potential test day and test order effects on the different treatment groups. Bees were released from the harness or holding tube into the testing arena and allowed to acclimatise for 1 minute. The 5 min test began 13–15 minutes after topical application and approximately 30 minutes after injection through the median ocellus.

The arena has been described previously [47]. Briefly, two side chambers (one containing nestmates (NM) and the other containing non-nestmates (NON)) flank the middle chamber where the test bee is placed. Mesh sides prevent bees moving between chambers, but the test bee can interact fully with the stimulus bees through the mesh. To begin testing, the sliding sides that separated the middle chamber from the two side chambers were removed, leaving only the mesh between the test bee and stimulus bees. Behavioural scoring was done live, including the frequency of dyadic interactions. A dyadic interaction is when the test bee and a stimulus bee rapidly antennate each other face-to-face for 2 seconds or more and/or trophallaxis occurred. An observer blind to treatments and which side chamber contained nestmates recorded sociability (amount of 5 min test time spent on the two mesh sides) and nestmate affiliation (proportion of time total time on the mesh sides spent on the NM mesh) from videos of each test. Stimulus bees were fed honey every 45 minutes to prevent hunger effects on interactions.

### Biogenic amine quantification

High performance liquid chromatography (HPLC) measurements of brain biogenic amine levels were used to examine neurophysiological effects of isolation as well as the effectiveness of treatment methods. Immediately after testing, bees were flash-frozen in liquid nitrogen and kept on dry ice until storage at -80°C. Heads were freeze-dried for 55 minutes at less than -35°C and under a pressure of less than 300mT (VirTis BenchTop K-series freezedryer). Whole brains (including the sub-oesophageal gland) were dissected from the head capsule on dry ice to keep the tissue frozen. Brains were then stored at -80°C. The amount of OA, DA and 5HT per brain was quantified using an Agilent 1200 series HPLC machine (Agilent Technologies, Sant Clare, CA, USA) connected to an electrochemical detector (ESA coulechem III) and dual electrode analytical cell (ESA 5011A, Chelmsford, MA, USA).

Methods for HPLC quantification are similar to Søvik et al., [59] and Nouvian et al., [26]. Briefly, for biogenic amine extraction from brain tissue, samples were removed from -80°C storage and centrifuged for 2 minutes at 13200rpm and 0°C. Kept cold on ice, 80μl of extraction solution (0.2 mol/L perchloric acid containing 10pg/μl 2,3-dihydroxybenzoic acid, DHBA (internal standard)) was added to each sample. Samples were then sonicated for 8 seconds to disrupt the tissue. Following sonication, samples were incubated in the dark at 0°C for 20 minutes, then centrifuged for 14 minutes (13200 rpm and 0°C). 70μl of the solution was loaded into the autosampler and 10μl was injected. 7-point standard curves of the external standards of OA, DA, 5HT and tyramine (all from Sigma-Aldrich, Australia) and the internal standard DHBA were run before and after each run of 24 samples. Nearly all tyramine measures of the samples did not reach detection levels and could not be analysed. Biogenic amine amounts were calculated from their peak area, which had been normalised to the size of the DHBA internal-standard peak within the same sample run, and quantified relative to the average of the two standard curves from before and after the sample run.

### Statistical analysis

All analyses were done using R (version 3.3.1) and confidence limits were set at 95% unless otherwise stated. All behavioural analyses were done on the data sets minus any data points that spent greater than 1 minute on their back.

#### Effects of topical treatment and isolation on brain biogenic amine levels

To confirm that the topical treatments were effective at introducing compounds to the brain, a two-way ANOVA was performed on the OA brain levels of all rearing-treatment groups. Treatment and rearing were explanatory variables. HPLC cannot detect the DA agonist, 6,7-ADTN, and bees were not treated with 5HT. Hence, a one-way ANOVA was done for each of these biogenic amines, with rearing group as the explanatory variable, to determine if isolation had an overall effect on DA and 5HT brain levels. One outlier for OA and two outliers for DA were removed from analysis (S1 Fig).

The frequency of the test bee interacting with a stimulus bee was converted to a binomial score of yes or no, and a likelihood (%) to interact calculated. A comparison of the likelihood of a dyadic interaction occurring between different treatment groups was performed using a *G*-test of independence and *post hoc* pairwise comparisons.

#### Effects of ocellar injection and isolation on brain biogenic amine levels

To confirm that ocellus injection was effective at introducing compounds to the brain, a two-way ANOVA with treatment and the rearing group as explanatory variables was performed on the DA, OA and 5HT brain levels of all rearing-treatment groups, except antagonist treatments as they were not given to isolated bees. Treatment and rearing were set as the explanatory variables so that a comparison of the hive-reared and isolated bees could also be done. All available brain data was used (**Table 2**). Any *post hoc* analyses were done using a Tukey HSD test.

Both sociability and nestmate affiliation behavioural data were not normally distributed thus non-parametric statistical methods were used. To explore potential rearing-treatment effects on sociability, a Kruskal-Wallis and Dunn’s (Holm-Bonferroni method) *post hoc* test was applied to all the behavioural data except hive-reared antagonist treatments. To determine if agonist and antagonist injection altered hive-reared bee sociability differently, a Mann-Whitney *U* test was used to compare each agonist-antagonist pair. A *G*-test of independence and *post hoc* pairwise comparisons determined if the likelihood of a dyadic interaction differed between rearing-treatment groups.

To determine if a preference to affiliate with nestmates (NM) over non-nestmates (NON) was expressed, each rearing-treatment group had their time spent on the NM mesh compared to their time spent on the NON mesh by a Wilcoxon signed-rank test. A Kruskal-Wallis test followed by a Dunn’s (Holm-Bonferroni method) *post hoc* test was then done to compare the proportion of time spent on the NM mesh by each rearing-treatment group except the hive-reared antagonist groups. A Mann-Whitney *U* test was used to compare each agonist-antagonist pair. For all statistical tests on nestmate affiliation, any bees that spent no time on either mesh or only visited one mesh side were removed in case they had not made a choice.

## Results

### Thoracic topical treatment elevated brain OA levels

OA brain levels were higher in OA-treated bees compared to all other treatment groups (**Table 3**), confirming that topical treatment via the thorax resulted in the compounds reaching the brain, at least by the end of the 5 minute behaviour test. No treatment effect could be assessed for the DA agonist since the HPLC machine could not detect 6,7-ADTN. No bees were treated with 5HT.

**Table 3:** The ANOVA result for brain biogenic brain levels in the topical experiment.

Groups included	Amine	Factor	Df	F	P values
All HPLC data	OA	Treatment	6	4.254	**<0.001**
		Rearing	1	4.752	**<0.05**
		Treat*rear	6	0.603	0.727
All HPLC data	DA	Rearing	1	11.22	**≤0.001**
All HPLC data	5HT	Rearing	1	24.12	**<0.0001**

### Isolation reduced OA, DA and 5HT brain levels

Rearing also had an effect on OA brain titers with hive-reared bees having on average higher brain OA levels than isolated bees (**Table 3**). Both DA and 5HT levels were also higher in hive-reared bees compared to isolated bees (**Table 3**).

### dMF impacted honey bee mobility and interactions

A clear effect on isolated bee mobility was found for the vehicle control group receiving the solvent dMF (**Table 1**). 64% of isolated bees topically treated with dMF spent greater than 1 minute of the test on their back. Although fewer isolated bees that received treatments containing dMF spent greater than 1 minute of the time on their back (28–34%), it was still more than controls (14%).

**Fig 1:**
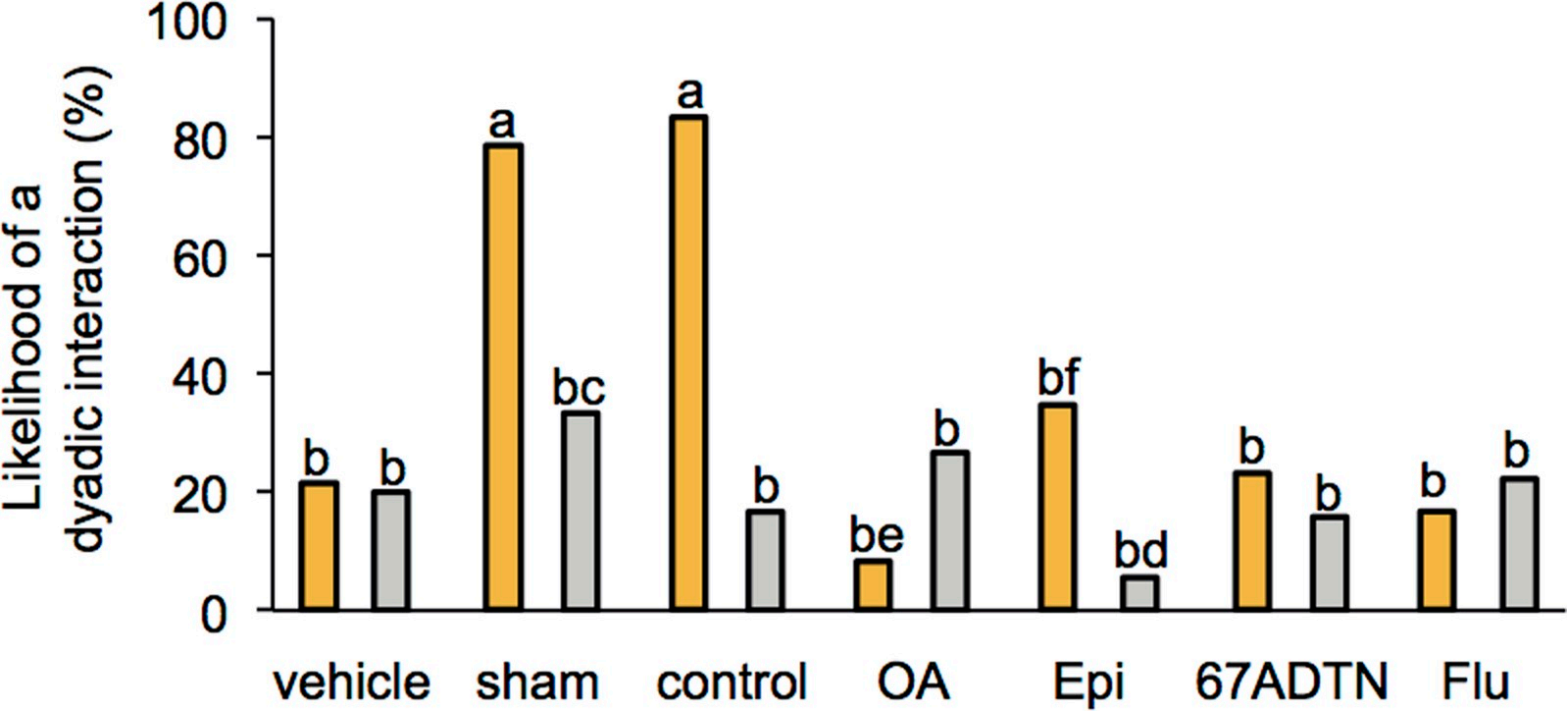
Solvent containing treatments reduced the rate of social interactions in hive-reared bees. The likelihood of hive-reared bees (gold) interacting with a stimulus bee was significantly reduced in treatment groups containing dMF (*G* = 57.10, df = 13, *P* < 0.0001; *pairwise comparisons*: a > b, *p* < 0.05–0.0001).

Moreover, all treatments containing dMF significantly reduced the likelihood of hive-reared bees interacting with the social stimuli (**Fig 1**, *G*-test of independence: *G* = 57.10, df = 13, *P* < 0.0001). Hive-reared bees normally engage in a social interaction more than isolated bees [17] but only the control and sham-control groups did so in the present study (**Fig 1**). Hence, no further analysis on the topical data was done.

### Ocellus injections of biogenic amines increased biogenic amine brain levels

To verify that ocellus injection of each biogenic amine resulted in higher levels of the respective amine in the brain, we compared the brain biogenic amine contents of each rearing-treatment group except hive-reared antagonists. Injection of DA and 5HT via the median ocellus increased their brain levels (**Table 4**), and OA injection was effective in hive-reared bees, but not in isolated bees (**Table 4**, Tukey HSD *post hoc*: (hive reared OA) hr-OA 581pg/brain ± 182.33 vs hr-control 295pg/brain ± 27.53, *p* = 0.05 and hr-OA vs hr-saline 269pg/brain ± 26.33, *p* < 0.05). Ocellus injection was also particularly effective for 5HT in hive-reared bees (**Table 4**, *post hoc*: hr-5HT 981pg/brain ± 121.66 vs hr-control 632pg/brain ± 53.55, *p* < 0.001, hr-5HT vs hr-saline 578pg/brain ± 53.55, *p* < 0.0001, and hr-5HT vs hr-sham 774pg/brain ± 53.55, *p* = 0.330).

**Table 4:** The two-way ANOVA results for brain biogenic amine levels for the ocellar injection experiment.

Amine	Factor	Df	F	P value
DA	Treatment	5	2.382	**<0.05**
	Rearing	1	4.235	**<0.05**
	Treat*Rear	5	1.259	0.283
OA	Treatment	5	1.117	0.353
	Rearing	1	0.253	0.616
	Treat*Rear	5	2.344	**<0.05**
5HT	Treatment	5	2.614	**<0.05**
	Rearing	1	0.694	0.406
	Treat*Rear	5	4.588	**<0.001**

Overall, hive-reared bees had higher brain levels than isolated bees for all three amines, but only significantly higher for DA (**Table 4**).

### Dopamine modulates honey bee sociability

**Fig 2:**
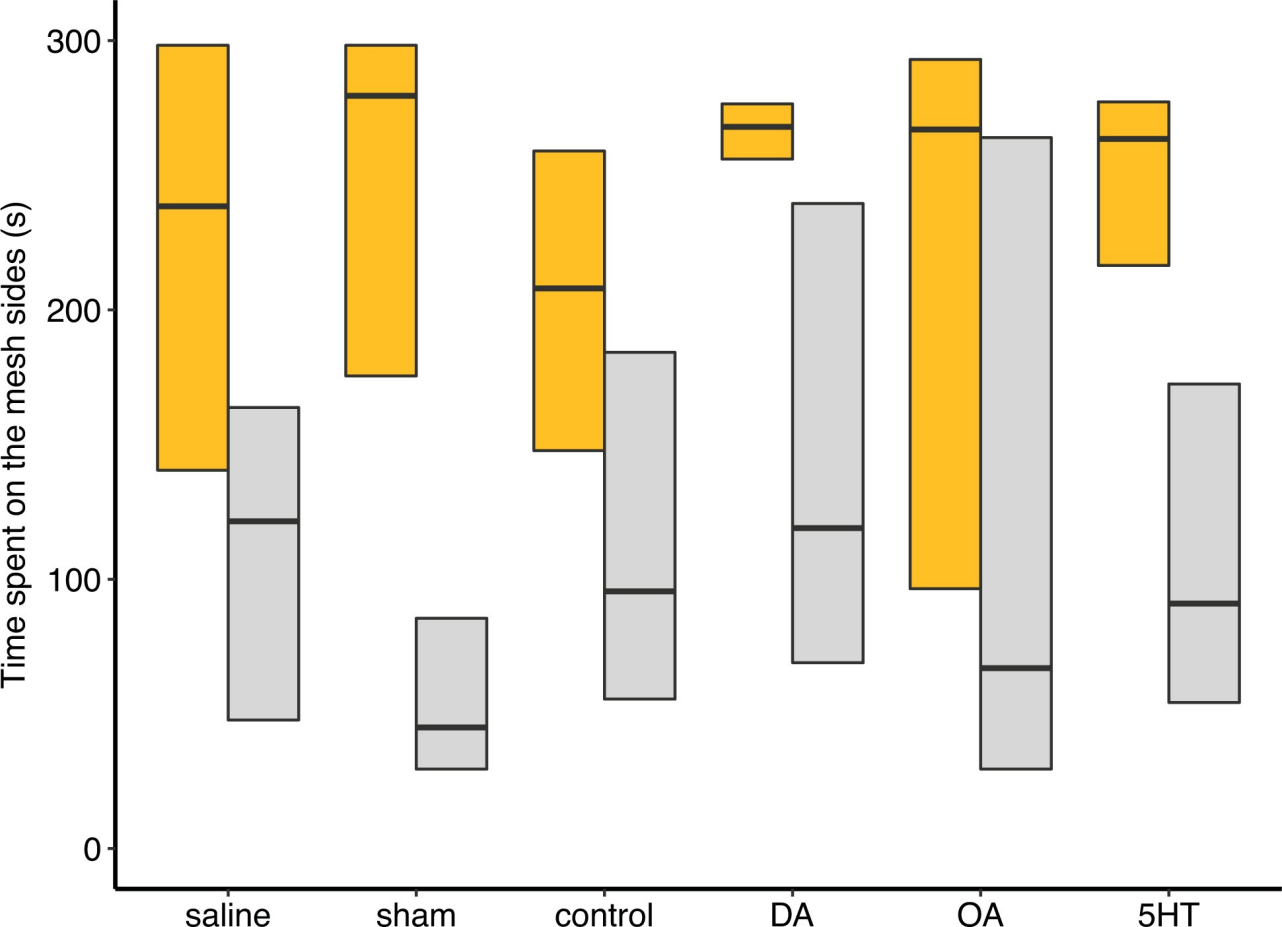
DA modulates honey bee sociability. Hive-reared bees (gold) injected with DA consistently remained on the mesh and in proximity to conspecifics for the majority of the test time. All isolated bee groups (grey) spent significantly less time interacting with the mesh, except the DA injected isolated group (Kruskal-Wallis: χ^2^ = 47.53, df = 11, *P* < 0.0001). OA injection increased the variation in sociability scores for both hive-reared and isolated bees and OA-iso was not significantly different to hr-DA (Dunn’s *post hoc* test: *p* = 0.063). Boxplots are the median and the 1^st^ and 3^rd^ quartiles.

**Fig 3:**
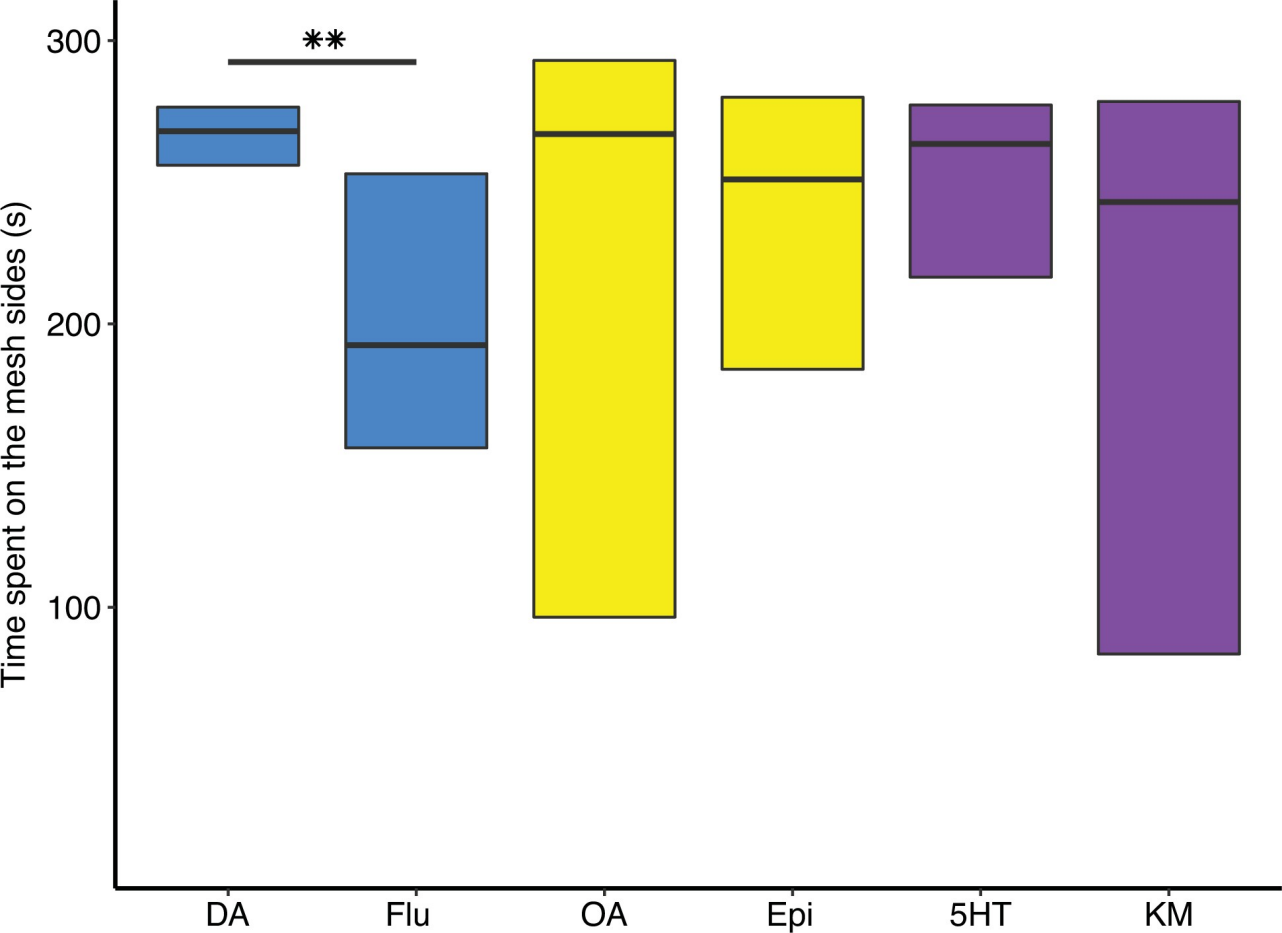
DA antagonist has opposing effect to DA injection. Hive-reared bees injected with DA and a DA antagonist fluphenazine (Blue boxes) differed in sociability scores (Mann-Whitney *U*: *W* = 195, *P = 0.01). OA and the antagonist epinastine (yellow boxes) did not differ in sociability scores (*W* = 117, *P* = 0.868), and neither did 5HT and the double 5HT antagonist treatment of ketanserin and methiothepin (purple boxes, *W* = 137, *P* = 0.527).

**Fig 4:**
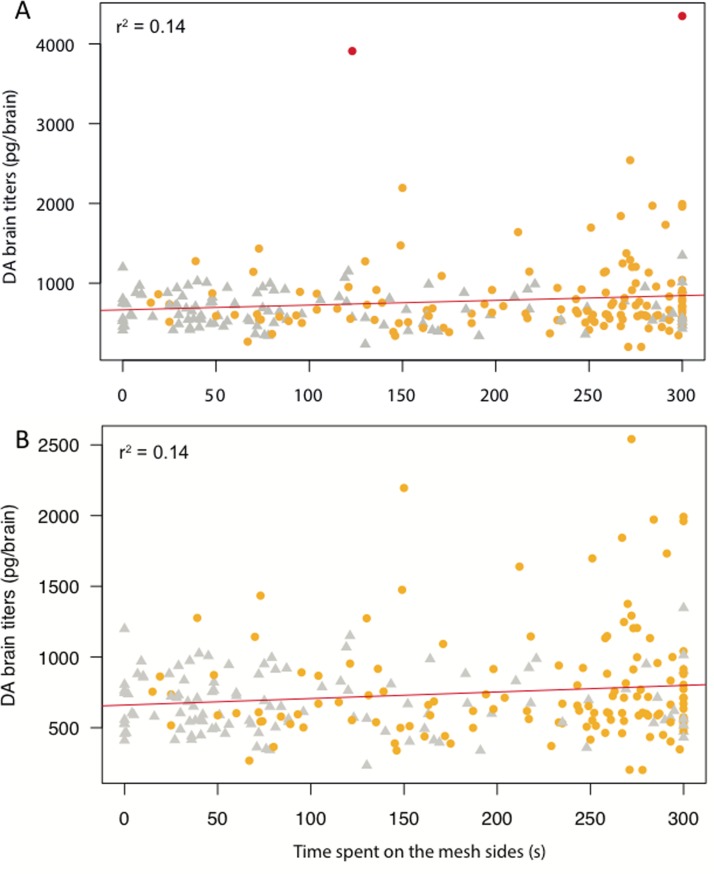
Sociability score positively correlates with DA brain levels. **A.** All brain-behaviour data available was plotted (gold circles = hive reared and grey triangles = isolated) and there is an overall positive correlation between time spent in proximity to conspecifics and DA brain titers (Pearson: *t* = 2.164, df = 245, *P* = 0.03). **B.** Removing the two high outlying DA values further increased the correlation (Pearson: *t* = 2.270, df = 243, *P* = 0.02).

The time spent on the mesh sides in proximity to stimulus bees was compared between each rearing-treatment group barring hive-reared-antagonist groups. A significant difference in median values was found across groups (Kruskal-Wallis: χ^2^ = 47.53, df = 11, *P* < 0.0001). DA injected hive-reared bees had high sociability scores (**Fig 2**), significantly higher than all isolated groups except DA injected isolated bees (**Fig 2**, Dunn’s *post hoc*: hr-DA vs (isolated DA) iso-DA, *p* = 0.273; hr-DA vs iso-OA, *p* = 0.06 and hr-DA vs iso-all others, *p* < 0.05–0.001), inferring an increase in sociability by DA treatment in both hive-reared and isolated bees. DA injected hive-reared bees were also significantly more sociable than hive-reared bees injected with a DA antagonist, fluphenazine (**Fig 3**, Mann Whitney *U* test: *W* = 195, *P* = 0.01) but not epinastine (*W* = 143, *P* = 0.374) which binds to both OA1α and DOP2 receptors in bees [34]. Furthermore, sociability score positively correlated with brain DA level when hive-reared and isolated data were combined (**Fig 4**, Pearson: t = 2.164, df = 245, *P* < 0.05).

### Effects of biogenic amine treatments on social interactions

Hive-reared bees injected with DA or 5HT were the most likely to interact with a stimulus bee, and no OA-injected hive-reared bees had a dyadic interaction (G-test of independence: *G* = 28.30, df = 11, *P* < 0.01). Comparing hive-reared biogenic amine and antagonist treatments also revealed differences in their effects (G-test of independence: *G* = 20.81, df = 5, *P* < 0.001). Epinastine injected bees were more likely to interact than OA-treated hive-reared bees (*post hoc* pairwise comparison: hr-Epi vs hr-OA, p < 0.05), and less likely to interact than DA-treated hive-reared bees (*post hoc*: hr-DA vs hr-Epi, p < 0.05). Fluphenazine and KM injected bees interacted less than their agonist pair (DA and 5HT, respectively) but not significantly.

### Possible effects of a DA antagonist on nestmate affiliation

**Fig 5:**
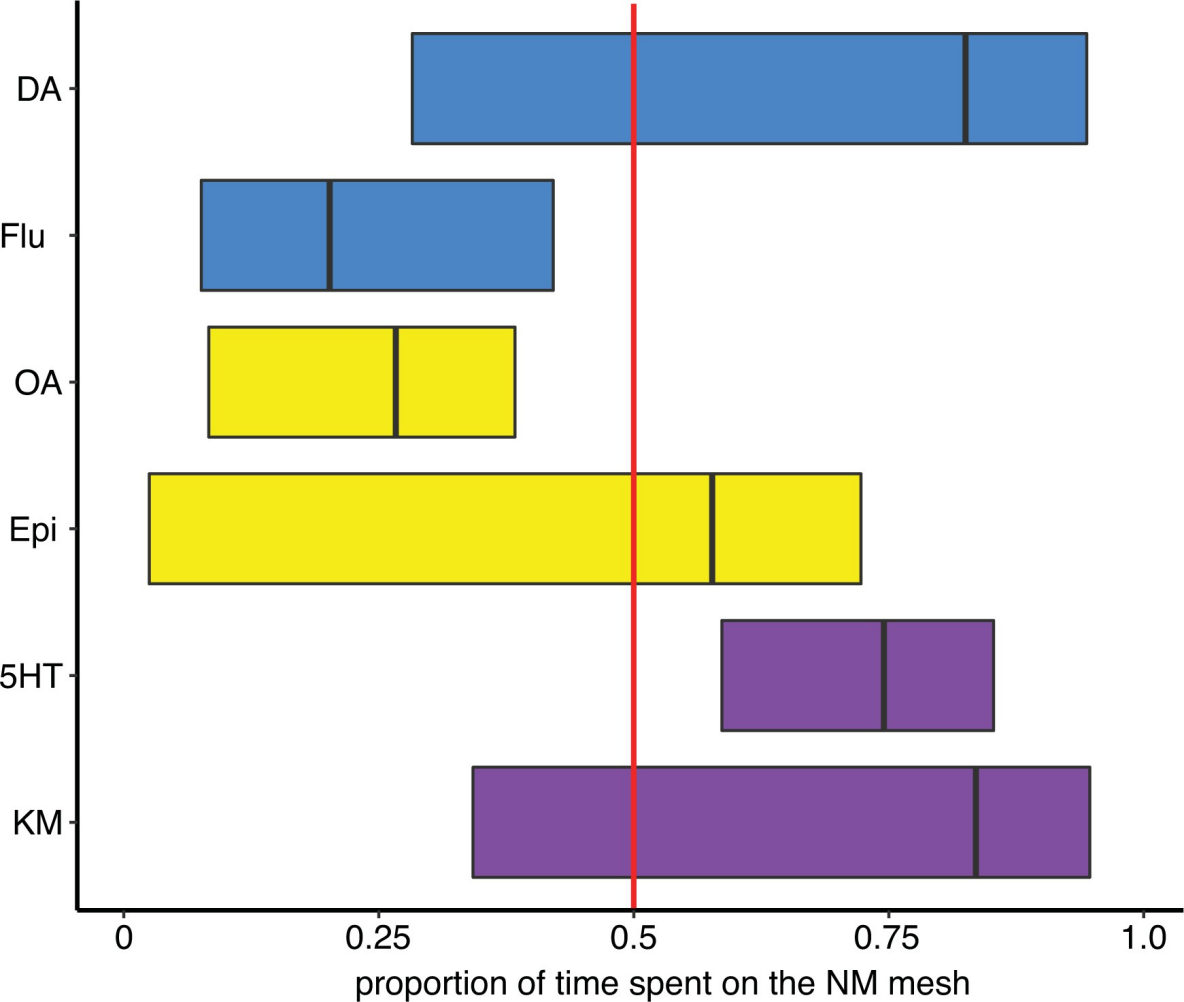
DA antagonists promote an affiliation to non-nestmates. Fluphenazine significantly increased the proportion of time spent with NON when compared to DA-injected bees (blue boxes, Mann Whitney *U* test: *W* = 127, *P* < 0.05). Neither OA or 5HT treatments differed from their antagonist treatments (yellow boxes, OA: *W* = 45, *P* = 0.766 and purple boxes, 5HT: *W* = 66, *P* = 0.538). The red line marks the same amount of time spent with NM and NON.

Overall no rearing-treatment groups expressed a significant preference for NM or NON in this assay. Comparing the time spent on the NM mesh by each rearing-treatment group (except hive-reared antagonist treatments) also revealed no significant differences between the median scores (Kruskal-Wallis: χ^2^ = 16.28, df = 11, *P* = 0.131). However, comparing each agonist-antagonist treatment pair showed that fluphenazine significantly increased the time spent with NON compared to DA-injected bees (**Fig 5**, Mann Whitney *U* test: *W* = 127, *P* < 0.05). Neither OA or 5HT treatments differed from their coupled antagonist treatments (**Fig 5**, OA: *W* = 45, *P* = 0.766 and 5HT: *W* = 66, *P* = 0.538).

## Discussion

### Topical treatment

We successfully increased honey bee brain amine titers by topical application on the thorax, as evidenced by the OA brain titer data (Table 3). The method has previously proven effective [50] and is often preferred in studies using freely moving insects [26, 55]. Unfortunately, we found that the solvent required to transport the biogenic amines and receptor antagonists across the cuticle impaired the mobility of 5 day old bees isolated since eclosion (Table 1), and reduced the likelihood of normally raised bees interacting with stimulus bees (Fig 1). Unhindered movement and social interactions are important components of this assay, thus, our conclusions from the topical experiments were limited. As topical application of substances dissolved in dMF is used relatively frequently in insect studies, we felt it important to mention the deleterious effects on locomotion and behaviour we encountered in the present study.

Even so, analyses of the brain amine levels of bees from this study did show isolated bees had lower levels of all three measured biogenic amines when compared to hive reared bees. Similar findings have been reported for comparisons of the solitary and sociable states of swarming locust species [36], colony-reared and isolated eusocial ants [29–30], and group-raised and isolated-post-weaning mammals [60].

### Ocellar injection

Overall, hive-reared bees had higher DA brain levels than isolated bees (Table 4) and there was a positive correlation between sociability score and DA brain titers (Fig 4). Activation and blocking of the DA system had opposing effects on both sociability and nestmate affiliation in hive-reared bees that had developed in a normal social environment. DA injection promoted consistently high levels of sociability (Fig 2), and increased the likelihood of interactions. Blocking DOP2-like receptors facilitated a preference to affiliate with NON (Fig 5). DA activity may also be involved in initiating the approach response in a socially naïve bee (Fig 2). OA injection eliminated the likelihood of a dyadic interaction among hive-reared bees. 5HT had no effect on the sociability or NM affiliation of both hive-reared and isolated bees, but had the same positive effect as DA on the likelihood of having an interaction.

#### Dopamine modulates honey bee sociability and nestmate affiliation

The results of DA treatment are consistent with the positive correlation between brain DA level and sociability (Figs 2 and 4 and Table 4). A previous study found manipulation of the DA systems by injection reduced locomotion in bees due to increased stationary grooming or remaining motionless [61]. However, Mustard et al., [61] found the same change in activity when either DA or an antagonist was injected, whereas we had opposing effects, suggesting a different cause for our results. DA activity has also been connected to lowering arousal state in flies [62–63] and the bees could be ‘asleep’ on the mesh. However, in this study the increased time spent on the mesh sides coincided with a maximal rate of dyadic social interactions, implying the reduced mobility is due to increased sociability and not rest.

The role of DA in vertebrate brain reward systems is well established [64], and in honey bees there is evidence for a role of DA in aversive learning [57]. DA modulation is also implied in reward learning of flies in highly motivated states, such as thirst and hunger [19–20, 65]. Our data are consistent with the theory of insect sociability enlisting activity of the insect reward circuitry, and provide evidence for dopaminergic modulation in the honey bee. Alternatively, the social stimuli could be a secondary reinforcer to a food reward as bees feed each other by trophallaxis [66–67]. Both tactile and olfactory stimuli promote reward learning in bees [68], even after only one trophallactic event [69]. However, we removed any hunger effects by satiating all bees as this affects both behaviour and physiology [70], and found DA rather than OA (that has a prominent role in bee sucrose reward learning [19]) promoted the approach response to the social stimulus and increased the likelihood of a dyadic interaction, inferring a response to the conspecifics themselves.

Isolated bees injected with DA also expressed higher sociability levels than the other isolated bee groups, as their score did not differ from DA-injected hive-reared bees (Fig 2). Activation of specific DA receptor types is involved in the switch from a solitary to a sociable phenotype in *L*. *migratoria* without exposure to the triggering sensory cues [41], and DA may also be involved in triggering honey bee sociability. Furthermore, different DA receptors in the honey bee antennae correspond to different responses to queen mandibular pheromone, and are altered by early life exposure [32].

The hive-reared DA-injection group had a high rate of dyadic interactions, and as no aggressive interactions were reported in this assay, the increase caused by DA was on positive social interactions, fitting with the increased level of sociability. Blocking DOP2-like receptors may have increased interest in the novel bees (NON) or increased avoidance of the familiar social stimuli (NM), as bees treated with fluphenazine spent significantly more time with NON compared to DA treated bees (Fig 5). The first scenario fits better with the results of DA on sociability and absence of aggressive behaviour. There is a parallel here to findings in prairie vole partnerships, where D2 receptors specifically are activated during formation of a partner preference in both males and females [7–8].

There were no antagonist effects when compared to controls. This may be due to the receptor subtypes targeted by the antagonists. Fluphenazine blocks DOP2-like receptors in mammals [71] and the bee DOP2-like receptor, AmDOP3, is structurally related to vertebrate DOP2-like receptors [61, 72]. Epinastine binds to a bee DOP1-like receptor: AmDOP2, as well as the adrenergic OA receptor: AmOA1 [34]. However, bees have another DOP1-like receptor: AmDOP1, which may not have been inhibited in the antagonist treatments. Dop1 receptors are involved in the shift from solitary to sociable in *L*. *migratoria* [41], and may explain the lack of antagonist effects on bee sociability.

#### Octopamine injection blocked dyadic interactions

OA is implicated in several studies on social interaction behaviour in social insects [27, 28–30]. Although Robinson et al., [27] found a bidirectional effect of OA by increased aggression to NON and increased cohesion with NM, other studies tend to find OA predominantly increases aggression to unfamiliar conspecifics [28–30]. In this assay we found OA treatment of hive-reared bees led to no social interactions with stimulus bees. The reaction was towards both familiar and unfamiliar stimuli implying that in this assay any discriminatory processes were overridden by the OA signal to avoid contact. OA is similar to noradrenalin in vertebrates, the neurohormone that initiates the fight or flight response [73], and may also predominate control under stressful conditions in bees.

#### Minimal effects of serotonin injection

5HT injection in hive-reared bees increased the likelihood of social interactions, like DA-treatment. However, no other notable effects were found. The antagonist cocktail had significant effects on *S*. *gregaria* sociability at the same concentration [35] but not here on bee sociability and affiliation. However, Anstey et al., [35] injected the treatments peripherally and we injected centrally. The reduction in brain levels of 5HT caused by isolation encourages a revisit to a possible role of 5HT in bee sociability.

In summary, all three biogenic amines altered bee interactions with conspecifics in this assay. Although several studies have implicated cuticular hydrocarbons as major recognition cues in ants, work identifying such cues in bees remains inconclusive [74]. To prevent exclusion of potential discrimination cues, we used live NM and NON stimulus bees, separated from the focal bee by a single-layer mesh screen. Hence, determining the specific stimuli bees were reacting to when approaching NM or NON in the arena could be a productive direction for future research. The results of this study provide testable hypotheses that can be explored using circuit level neuro-imaging methods for further elaboration on the discrimination modalities used by bees, and whether reward system activation is involved.

## Conclusion

We report strong evidence for a causal role of DA in bee sociability and group cohesion, and corroborate findings connecting OA to eusocial insect social interactions. A strong possibility is a connection to brain reward systems. This study also illustrates the limitations of neuropharmacological manipulation experiments, either because of the application method or a lack of antagonists specific to receptor subtypes. Nonetheless, our findings provide a better overview of the neurochemical mechanisms driving sociability and group cohesion in eusocial insects.

## Supporting information

S1 Fig Individual brain levels of OA (A) and DA (B) used in the 2-way and 1-way ANOVA analyses. The black points and error bars are the mean ± S.E. Red points are the outliers removed for statistical analysis, gold points represent hive-reared and grey points are isolated bees.
(TIFF)
