# Supplementary figures and images for: Biogenic amine modulation of honey bee sociability and nestmate affiliation
Source: PLoS One. 2018 Oct 25;13(10):e0205686. doi: 10.1371/journal.pone.0205686 (PMC6201892; doi:10.1371/journal.pone.0205686)

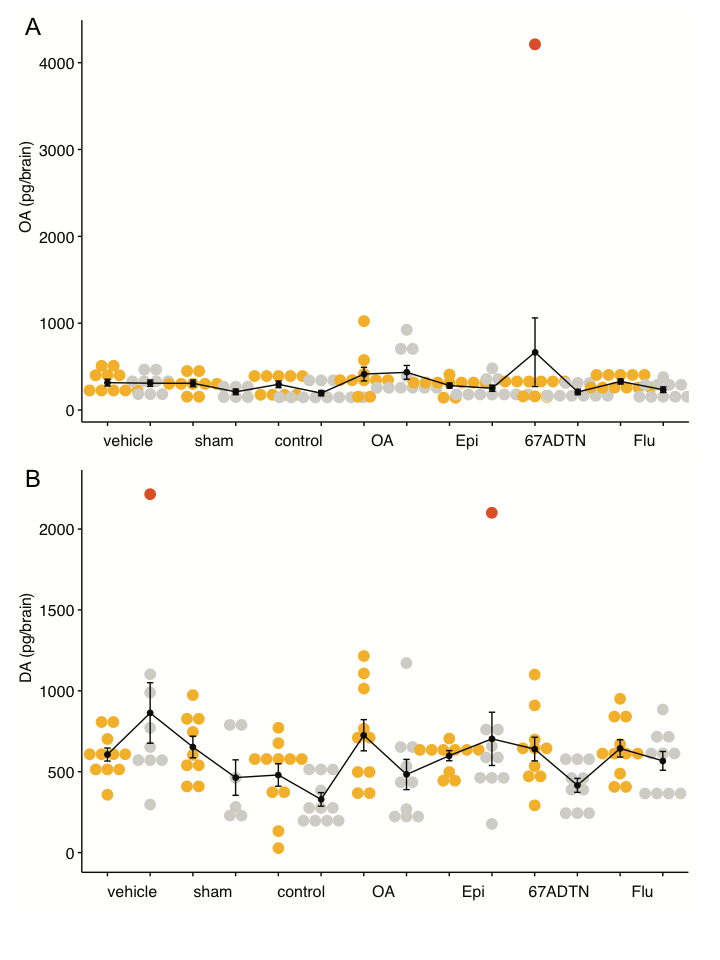

Supplement: S1 Fig — Individual brain levels of OA (A) and DA (B) used in the 2-way and 1-way ANOVA analyses. The black points and error bars are the mean ± S.E. Red points are the outliers removed for statistical analysis, gold points represent hive-reared and grey points are isolated bees. (TIFF) [file pone.0205686.s001.tiff]
